# Methyl 6-oxo-4-phenyl-2-[(*Z*)-2-(pyridin-2-yl)ethen­yl]-1,4,5,6-tetra­hydro­pyridine-3-carboxyl­ate

**DOI:** 10.1107/S1600536812048532

**Published:** 2012-11-30

**Authors:** Rufus Smits, Sergey Belyakov, Brigita Vigante, Gunars Duburs

**Affiliations:** a Latvian Institute of Organic Synthesis, Riga, LV 1006, Latvia

## Abstract

In the title mol­ecule, C_20_H_18_N_2_O_3_, an intra­molecular N—H⋯O hydrogen bond leads to a *cis* conformation of the pyridinyl-vinyl fragment. The phenyl and pyridine rings are inclined to one another by 77.3 (1) °. In the crystal, mol­ecules are linked *via* pairs of C—H⋯O hydrogen bonds, forming inversion dimers. The dimers are linked by C—H⋯O hydrogen bonds and C—H⋯π inter­actions, forming a three-dimensional structure.

## Related literature

For applications of dihydro­pyridones, see: Dong *et al.* (2005 ▶); Elias *et al.* (2008 ▶). For a description of the Cambridge Structural Database, see: Allen (2002 ▶).
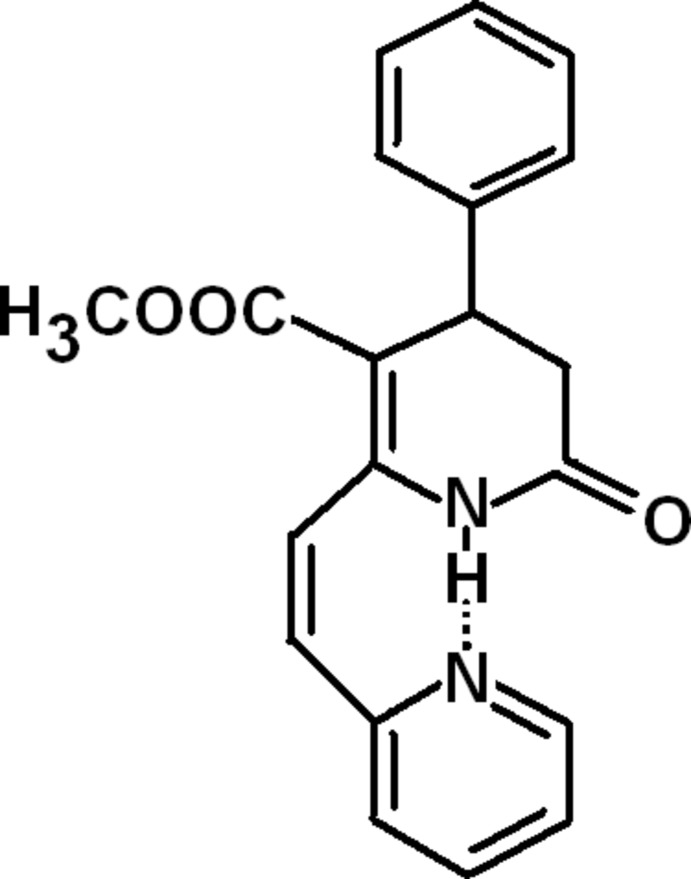


## Experimental

### 

#### Crystal data

C_20_H_18_N_2_O_3_
*M* *_r_* = 334.36
Monoclinic, 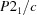

*a* = 5.5746 (2) Å
*b* = 16.4083 (6) Å
*c* = 18.0930 (8) Å
β = 96.5018 (14)°
*V* = 1644.32 (11) Å^3^
*Z* = 4
Mo *K*α radiation
μ = 0.09 mm^−1^
*T* = 193 K
0.41 × 0.12 × 0.07 mm

#### Data collection

Bruker–Nonius KappaCCD diffractometer
7024 measured reflections
4206 independent reflections
2483 reflections with *I* > 2σ(*I*)
*R*_int_ = 0.053

#### Refinement

*R*[*F* ^2^ > 2σ(*F* ^2^)] = 0.060
*wR*(*F* ^2^) = 0.149
*S* = 0.96
4206 reflections
242 parameters
H atoms treated by a mixture of independent and constrained refinement
Δρ_max_ = 0.23 e Å^−3^
Δρ_min_ = −0.22 e Å^−3^

### 

Data collection: *KappaCCD Server Software* (Nonius, 1999 ▶); cell refinement: *DENZO* and *SCALEPACK* (Otwinowski & Minor, 1997 ▶); data reduction: *DENZO* and *SCALEPACK*; program(s) used to solve structure: *SHELXS97* (Sheldrick, 2008 ▶); program(s) used to refine structure: *SHELXL97* (Sheldrick, 2008 ▶); molecular graphics: *ORTEP-3* (Farrugia, 1997 ▶); software used to prepare material for publication: *maXus* (Mackay *et al.*, 1999 ▶).

## Supplementary Material

Crystal structure: contains datablock(s) global, I. DOI: 10.1107/S1600536812048532/cv5365sup1.cif

Structure factors: contains datablock(s) I. DOI: 10.1107/S1600536812048532/cv5365Isup2.hkl

Supplementary material file. DOI: 10.1107/S1600536812048532/cv5365Isup3.cml

Additional supplementary materials:  crystallographic information; 3D view; checkCIF report

## Figures and Tables

**Table 1:** Hydrogen-bond geometry (Å, °) *Cg* is the centroid of the C8–C13 phenyl ring.

*D*—H⋯*A*	*D*—H	H⋯*A*	*D*⋯*A*	*D*—H⋯*A*
C25—H25⋯O15^i^	0.93	2.42	3.257 (3)	150
C24—H24⋯O7^ii^	0.93	2.49	3.318 (3)	149
C13—H13⋯O16^iii^	0.93	2.54	3.300 (3)	139
C23—H23⋯*Cg* ^ii^	0.93	2.66	3.480	147

Symmetry codes: (i) 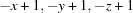; (ii) 
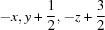
; (iii) 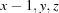.

**Fig. 1.**
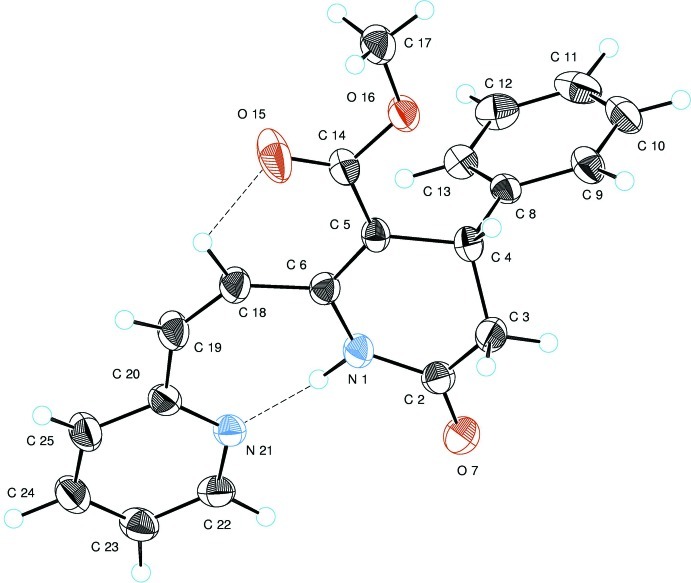
The molecular structure of (I) showing 50% probability displacement ellipsoids and the atom numbering scheme. Dashed lines denote intramolecular hydrogen bonds.

## supplementary crystallographic information

### Comment

Dihydropyridones are important intermediates for the synthesis of natural products, particularly alkaloids (Dong *et al.*, 2005; Elias *et al.*, 2008). They have been extensively investigated as valuable building blocks for the construction of piperidines, perhydroquinolines, indolizidines, quinolizidines and other alkaloid systems, with a wide range of biological and pharmacological activities. Herewith we present the title compound (I).

The main feature of (I) (Fig. 1) is *cis*-conformation of the pyridinylvinyl fragment, see Table 1 for selected torsion angles. A search of the Cambridge Structural Database (CSD, Version 5.33; November, 2012) (Allen, 2002) indicates that there is no entry containing pyridinylvinyl substituent in *cis*-conformation. Molecular *cis*-conformation is stabilized by strong intramolecular hydrogen bond of NH···N type (Table 2). By means of this bond the additional seven-membered cycle is formed in the molecular structure. In the molecule there is also an intramolecular hydrogen bond of CH···O type (Table 2). This bond leads to formation of the additional six-membered cycle in the molecule.

In the crystal structure there are shortened C···O contacts. These contacts can be described as weak CH···O type intermolecular hydrogen bonds. Also it should be noted a weak CH···π type H-bond. The geometrical parameters of these H-bonds are given in Table 2.

### Experimental

In a 50 ml RB was placed 0.59 g (0.001 mol) of the DHPOD 6-methyltriphenylphosphonium bromide and dissolved in 25 ml dry THF. Under an Ar atmosphere while stirring magnetically 0.22 g (0.001 mol) of tBuOK was added. The orange solution was stirred for 30 min and 0.11 g (0.001 mol) of 2-pyridinecarboxaldehyde was added. The solution was allowed to stir at RT overnight, 3 ml of aqueous solution containing 0.6 g NH_4_Cl was added and after stirring 15 min the layers were separated. The THF was removed under reduced pressure and the sticky reaction product was dissolved in min. EtOAc. After addition of hexane the precipitated triphenylphosphine oxide was filtered off and the solvent removed to leave 0.55 g of product. The product was purified using prep. HPLC with 50% EtOAc / DCM as eluent. The solvent was removed providing 0.21 g of product (62% yield) which was recrystallized from EtOH giving 100 mg of light green needles.

### Refinement

Atoms H1, H4, H18 and H19 were located on a difference map and isotropically refined. All other H-atoms were positioned geometrically (C—H = 0.93–0.97 Å) and allowed to ride on their parent atoms, with *U*_iso_(H) = 1.2 *U*_eq_(parent atom).

### Crystal data

**Table:**

C_20_H_18_N_2_O_3_	*D*_x_ = 1.351 Mg m^−^^3^
*M**_r_* = 334.36	Melting point: 504 K
Monoclinic, *P*2_1_/*c*	Mo *K*α radiation, λ = 0.71073 Å
*a* = 5.5746 (2) Å	Cell parameters from 2317 reflections
*b* = 16.4083 (6) Å	θ = 0.9–27.5°
*c* = 18.0930 (8) Å	µ = 0.09 mm^−^^1^
β = 96.5018 (14)°	*T* = 193 K
*V* = 1644.32 (11) Å^3^	Needle, colourless
*Z* = 4	0.41 × 0.12 × 0.07 mm
*F*(000) = 704	

### Data collection

**Table:**

Bruker–Nonius KappaCCD diffractometer	2483 reflections with *I* > 2σ(*I*)
Radiation source: fine-focus sealed tube	*R*_int_ = 0.053
Graphite monochromator	θ_max_ = 28.6°, θ_min_ = 1.6°
φ and ω scan	*h* = −7→7
7024 measured reflections	*k* = −20→22
4206 independent reflections	*l* = −24→24

### Refinement

**Table:**

Refinement on *F*^2^	Primary atom site location: structure-invariant direct methods
Least-squares matrix: full	Secondary atom site location: difference Fourier map
*R*[*F*^2^ > 2σ(*F*^2^)] = 0.060	H atoms treated by a mixture of independent and constrained refinement
*wR*(*F*^2^) = 0.149	*w* = 1/[σ^2^(*F*_o_^2^) + (0.0562*P*)^2^ + 0.7901*P*] where *P* = (*F*_o_^2^ + 2*F*_c_^2^)/3
*S* = 0.96	(Δ/σ)_max_ = 0.019
4206 reflections	Δρ_max_ = 0.23 e Å^−^^3^
242 parameters	Δρ_min_ = −0.22 e Å^−^^3^
0 restraints	

### Special details

**Table:**

Geometry. All e.s.d.'s (except the e.s.d. in the dihedral angle between two l.s. planes) are estimated using the full covariance matrix. The cell e.s.d.'s are taken into account individually in the estimation of e.s.d.'s in distances, angles and torsion angles; correlations between e.s.d.'s in cell parameters are only used when they are defined by crystal symmetry. An approximate (isotropic) treatment of cell e.s.d.'s is used for estimating e.s.d.'s involving l.s. planes.
Refinement. Refinement of *F*^2^ against ALL reflections. The weighted *R*-factor *wR* and goodness of fit *S* are based on *F*^2^, conventional *R*-factors *R* are based on *F*, with *F* set to zero for negative *F*^2^. The threshold expression of *F*^2^ > σ(*F*^2^) is used only for calculating *R*-factors(gt) *etc*. and is not relevant to the choice of reflections for refinement. *R*-factors based on *F*^2^ are statistically about twice as large as those based on *F*, and *R*- factors based on ALL data will be even larger.

### Fractional atomic coordinates and isotropic or equivalent isotropic displacement parameters (Å^2^)

**Table:**

	*x*	*y*	*z*	*U*_iso_*/*U*_eq_	
N1	0.3841 (3)	0.31397 (11)	0.68159 (10)	0.0317 (4)	
H1	0.249 (4)	0.3452 (16)	0.6900 (13)	0.050 (7)*	
C2	0.4865 (4)	0.26644 (13)	0.73871 (12)	0.0343 (5)	
C3	0.7199 (4)	0.22630 (13)	0.72613 (12)	0.0349 (5)	
H3A	0.7385	0.1771	0.7559	0.042*	
H3B	0.8519	0.2626	0.7433	0.042*	
C4	0.7383 (3)	0.20413 (12)	0.64446 (11)	0.0281 (5)	
H4	0.910 (4)	0.1941 (13)	0.6403 (11)	0.030 (5)*	
C5	0.6556 (3)	0.27567 (12)	0.59458 (11)	0.0275 (4)	
C6	0.4830 (3)	0.32727 (12)	0.61506 (11)	0.0281 (4)	
O7	0.3935 (3)	0.26137 (10)	0.79683 (9)	0.0471 (4)	
C8	0.6075 (3)	0.12466 (12)	0.62263 (11)	0.0266 (4)	
C9	0.7212 (4)	0.05100 (13)	0.64153 (13)	0.0357 (5)	
H9	0.8757	0.0510	0.6671	0.043*	
C10	0.6078 (4)	−0.02244 (14)	0.62283 (14)	0.0429 (6)	
H10	0.6862	−0.0712	0.6360	0.052*	
C11	0.3794 (4)	−0.02351 (14)	0.58485 (13)	0.0442 (6)	
H11	0.3044	−0.0728	0.5715	0.053*	
C12	0.2630 (4)	0.04892 (15)	0.56682 (13)	0.0411 (5)	
H12	0.1078	0.0484	0.5418	0.049*	
C13	0.3754 (4)	0.12274 (13)	0.58566 (12)	0.0327 (5)	
H13	0.2945	0.1713	0.5734	0.039*	
C14	0.7626 (4)	0.28495 (13)	0.52436 (12)	0.0320 (5)	
O15	0.7451 (4)	0.34235 (11)	0.48210 (12)	0.0704 (6)	
O16	0.8913 (3)	0.21953 (9)	0.50878 (8)	0.0371 (4)	
C17	1.0023 (4)	0.22380 (15)	0.44055 (13)	0.0441 (6)	
H17A	1.0904	0.1745	0.4344	0.053*	
H17B	1.1107	0.2694	0.4426	0.053*	
H17C	0.8795	0.2304	0.3993	0.053*	
C18	0.3926 (4)	0.39772 (13)	0.57107 (13)	0.0357 (5)	
H18	0.468 (4)	0.4026 (15)	0.5270 (14)	0.043 (6)*	
C19	0.2314 (4)	0.45687 (14)	0.58015 (13)	0.0378 (5)	
H19	0.219 (4)	0.4957 (15)	0.5424 (14)	0.045 (7)*	
C20	0.0609 (4)	0.47713 (12)	0.63325 (12)	0.0323 (5)	
N21	0.0217 (3)	0.42656 (10)	0.68919 (10)	0.0313 (4)	
C22	−0.1441 (4)	0.44794 (13)	0.73342 (12)	0.0348 (5)	
H22	−0.1708	0.4131	0.7722	0.042*	
C23	−0.2784 (4)	0.51884 (14)	0.72480 (13)	0.0398 (5)	
H23	−0.3942	0.5307	0.7564	0.048*	
C24	−0.2370 (4)	0.57122 (14)	0.66859 (14)	0.0429 (6)	
H24	−0.3229	0.6197	0.6616	0.052*	
C25	−0.0651 (4)	0.55059 (14)	0.62245 (13)	0.0400 (5)	
H25	−0.0333	0.5855	0.5843	0.048*	

### Atomic displacement parameters (Å^2^)

**Table:**

	*U*^11^	*U*^22^	*U*^33^	*U*^12^	*U*^13^	*U*^23^
N1	0.0379 (9)	0.0272 (9)	0.0312 (10)	0.0031 (8)	0.0090 (7)	0.0037 (8)
C2	0.0473 (12)	0.0256 (11)	0.0303 (12)	−0.0044 (9)	0.0058 (9)	−0.0001 (9)
C3	0.0420 (12)	0.0286 (11)	0.0321 (12)	−0.0019 (9)	−0.0040 (9)	0.0029 (9)
C4	0.0249 (10)	0.0252 (10)	0.0338 (12)	0.0011 (8)	0.0016 (8)	0.0029 (9)
C5	0.0270 (9)	0.0234 (10)	0.0316 (11)	−0.0020 (8)	0.0013 (8)	0.0019 (8)
C6	0.0302 (10)	0.0258 (10)	0.0284 (11)	−0.0023 (8)	0.0031 (8)	0.0019 (8)
O7	0.0687 (11)	0.0392 (9)	0.0355 (9)	0.0043 (8)	0.0157 (8)	0.0077 (7)
C8	0.0288 (10)	0.0251 (10)	0.0273 (10)	0.0023 (8)	0.0097 (8)	0.0012 (8)
C9	0.0344 (11)	0.0308 (11)	0.0437 (13)	0.0054 (9)	0.0116 (9)	0.0019 (10)
C10	0.0577 (15)	0.0250 (11)	0.0505 (15)	0.0047 (10)	0.0256 (12)	0.0005 (10)
C11	0.0577 (15)	0.0345 (13)	0.0444 (14)	−0.0161 (11)	0.0233 (12)	−0.0118 (11)
C12	0.0380 (12)	0.0459 (14)	0.0397 (13)	−0.0108 (11)	0.0061 (9)	−0.0067 (11)
C13	0.0313 (10)	0.0321 (11)	0.0348 (12)	−0.0009 (9)	0.0045 (8)	−0.0003 (9)
C14	0.0327 (10)	0.0263 (11)	0.0377 (12)	0.0021 (9)	0.0074 (9)	0.0029 (9)
O15	0.0974 (15)	0.0479 (11)	0.0770 (14)	0.0346 (10)	0.0584 (12)	0.0312 (10)
O16	0.0439 (8)	0.0318 (8)	0.0377 (9)	0.0096 (7)	0.0129 (7)	0.0041 (7)
C17	0.0530 (14)	0.0389 (13)	0.0432 (14)	0.0117 (11)	0.0183 (11)	0.0049 (11)
C18	0.0422 (12)	0.0329 (12)	0.0339 (13)	0.0065 (10)	0.0132 (10)	0.0072 (10)
C19	0.0449 (12)	0.0332 (12)	0.0375 (13)	0.0080 (10)	0.0139 (10)	0.0106 (11)
C20	0.0379 (11)	0.0279 (11)	0.0316 (12)	−0.0015 (9)	0.0069 (9)	−0.0019 (9)
N21	0.0373 (9)	0.0262 (9)	0.0315 (10)	−0.0027 (7)	0.0081 (7)	−0.0007 (7)
C22	0.0419 (11)	0.0319 (11)	0.0319 (12)	−0.0051 (10)	0.0098 (9)	−0.0028 (9)
C23	0.0431 (12)	0.0377 (13)	0.0406 (13)	0.0009 (10)	0.0142 (10)	−0.0060 (11)
C24	0.0520 (13)	0.0318 (12)	0.0470 (15)	0.0113 (10)	0.0141 (11)	0.0005 (11)
C25	0.0511 (13)	0.0293 (11)	0.0418 (13)	0.0069 (10)	0.0143 (10)	0.0056 (10)

### Geometric parameters (Å, º)

**Table:**

N1—C2	1.367 (3)	C12—H12	0.9300
N1—C6	1.397 (3)	C13—H13	0.9300
N1—H1	0.94 (3)	C14—O15	1.210 (3)
C2—O7	1.227 (3)	C14—O16	1.339 (2)
C2—C3	1.498 (3)	O16—C17	1.444 (3)
C3—C4	1.537 (3)	C17—H17A	0.9600
C3—H3A	0.9700	C17—H17B	0.9600
C3—H3B	0.9700	C17—H17C	0.9600
C4—C5	1.520 (3)	C18—C19	1.345 (3)
C4—C8	1.524 (3)	C18—H18	0.95 (2)
C4—H4	0.98 (2)	C19—C20	1.465 (3)
C5—C6	1.364 (3)	C19—H19	0.93 (3)
C5—C14	1.471 (3)	C20—N21	1.346 (3)
C6—C18	1.460 (3)	C20—C25	1.398 (3)
C8—C13	1.388 (3)	N21—C22	1.337 (3)
C8—C9	1.389 (3)	C22—C23	1.383 (3)
C9—C10	1.385 (3)	C22—H22	0.9300
C9—H9	0.9300	C23—C24	1.371 (3)
C10—C11	1.377 (3)	C23—H23	0.9300
C10—H10	0.9300	C24—C25	1.382 (3)
C11—C12	1.375 (3)	C24—H24	0.9300
C11—H11	0.9300	C25—H25	0.9300
C12—C13	1.388 (3)		
			
C2—N1—C6	124.69 (18)	C11—C12—H12	119.7
C2—N1—H1	117.6 (15)	C13—C12—H12	119.7
C6—N1—H1	117.2 (15)	C8—C13—C12	120.6 (2)
O7—C2—N1	120.4 (2)	C8—C13—H13	119.7
O7—C2—C3	124.0 (2)	C12—C13—H13	119.7
N1—C2—C3	115.55 (19)	O15—C14—O16	119.9 (2)
C2—C3—C4	113.83 (17)	O15—C14—C5	127.9 (2)
C2—C3—H3A	108.8	O16—C14—C5	112.22 (17)
C4—C3—H3A	108.8	C14—O16—C17	115.65 (17)
C2—C3—H3B	108.8	O16—C17—H17A	109.5
C4—C3—H3B	108.8	O16—C17—H17B	109.5
H3A—C3—H3B	107.7	H17A—C17—H17B	109.5
C5—C4—C8	113.75 (16)	O16—C17—H17C	109.5
C5—C4—C3	109.83 (17)	H17A—C17—H17C	109.5
C8—C4—C3	111.70 (16)	H17B—C17—H17C	109.5
C5—C4—H4	108.5 (12)	C19—C18—C6	134.3 (2)
C8—C4—H4	106.1 (12)	C19—C18—H18	114.0 (15)
C3—C4—H4	106.6 (12)	C6—C18—H18	111.6 (15)
C6—C5—C14	122.53 (18)	C18—C19—C20	137.4 (2)
C6—C5—C4	119.60 (18)	C18—C19—H19	113.4 (15)
C14—C5—C4	117.85 (17)	C20—C19—H19	109.1 (15)
C5—C6—N1	119.77 (18)	N21—C20—C25	121.03 (19)
C5—C6—C18	123.61 (19)	N21—C20—C19	121.74 (19)
N1—C6—C18	116.62 (18)	C25—C20—C19	117.21 (19)
C13—C8—C9	118.24 (19)	C22—N21—C20	118.07 (18)
C13—C8—C4	122.47 (17)	N21—C22—C23	123.8 (2)
C9—C8—C4	119.28 (17)	N21—C22—H22	118.1
C10—C9—C8	120.9 (2)	C23—C22—H22	118.1
C10—C9—H9	119.5	C24—C23—C22	118.5 (2)
C8—C9—H9	119.5	C24—C23—H23	120.8
C11—C10—C9	120.3 (2)	C22—C23—H23	120.8
C11—C10—H10	119.9	C23—C24—C25	118.8 (2)
C9—C10—H10	119.9	C23—C24—H24	120.6
C12—C11—C10	119.5 (2)	C25—C24—H24	120.6
C12—C11—H11	120.3	C24—C25—C20	119.9 (2)
C10—C11—H11	120.3	C24—C25—H25	120.1
C11—C12—C13	120.5 (2)	C20—C25—H25	120.1
			
C6—N1—C2—O7	176.29 (19)	C10—C11—C12—C13	0.9 (3)
C6—N1—C2—C3	−0.7 (3)	C9—C8—C13—C12	−1.4 (3)
O7—C2—C3—C4	151.0 (2)	C4—C8—C13—C12	179.99 (19)
N1—C2—C3—C4	−32.2 (3)	C11—C12—C13—C8	0.4 (3)
C2—C3—C4—C5	46.1 (2)	C6—C5—C14—O15	11.9 (4)
C2—C3—C4—C8	−81.1 (2)	C4—C5—C14—O15	−169.8 (2)
C8—C4—C5—C6	95.1 (2)	C6—C5—C14—O16	−167.78 (17)
C3—C4—C5—C6	−30.9 (2)	C4—C5—C14—O16	10.6 (2)
C8—C4—C5—C14	−83.3 (2)	O15—C14—O16—C17	0.2 (3)
C3—C4—C5—C14	150.69 (18)	C5—C14—O16—C17	179.93 (18)
C14—C5—C6—N1	178.39 (18)	C5—C6—C18—C19	−178.3 (2)
C4—C5—C6—N1	0.1 (3)	N1—C6—C18—C19	1.3 (4)
C14—C5—C6—C18	−1.9 (3)	C6—C18—C19—C20	−4.9 (5)
C4—C5—C6—C18	179.75 (18)	C18—C19—C20—N21	−5.5 (4)
C2—N1—C6—C5	18.1 (3)	C18—C19—C20—C25	176.1 (3)
C2—N1—C6—C18	−161.56 (19)	C25—C20—N21—C22	1.1 (3)
C5—C4—C8—C13	−27.0 (3)	C19—C20—N21—C22	−177.3 (2)
C3—C4—C8—C13	98.1 (2)	C20—N21—C22—C23	0.3 (3)
C5—C4—C8—C9	154.43 (19)	N21—C22—C23—C24	−1.2 (3)
C3—C4—C8—C9	−80.6 (2)	C22—C23—C24—C25	0.7 (3)
C13—C8—C9—C10	1.1 (3)	C23—C24—C25—C20	0.6 (4)
C4—C8—C9—C10	179.8 (2)	N21—C20—C25—C24	−1.6 (3)
C8—C9—C10—C11	0.1 (3)	C19—C20—C25—C24	176.9 (2)
C9—C10—C11—C12	−1.2 (3)		

### Hydrogen-bond geometry (Å, º)

Cg is the centroid of the C8–C13 phenyl ring.

**Table:**

*D*—H···*A*	*D*—H	H···*A*	*D*···*A*	*D*—H···*A*
C25—H25···O15^i^	0.93	2.42	3.257 (3)	150
C24—H24···O7^ii^	0.93	2.49	3.318 (3)	149
C13—H13···O16^iii^	0.93	2.54	3.300 (3)	139
C19—H19···O15^i^	0.93 (3)	2.71 (3)	3.489 (3)	142 (2)
C23—H23···*Cg*^ii^	0.93	2.66	3.480	147

Symmetry codes: (i) −*x*+1, −*y*+1, −*z*+1; (ii) −*x*, *y*+1/2, −*z*+3/2; (iii) *x*−1, *y*, *z*.
